# MiRNA Expression in Long-Distance Runners with Musculoskeletal Pain: Implications for Pain Pathophysiology

**DOI:** 10.3390/biomedicines12112494

**Published:** 2024-10-30

**Authors:** Maria Rosaria Tumolo, Antonella Bodini, Francesco Bagordo, Carlo Giacomo Leo, Pierpaolo Mincarone, Elisabetta De Matteis, Saverio Sabina, Tiziana Grassi, Egeria Scoditti

**Affiliations:** 1 Department of Biological & Environmental Sciences & Technology, University of Salento, 73100 Lecce, Italy; mariarosaria.tumolo@unisalento.it; 2 Branch of Lecce, National Research Council, Institute of Clinical Physiology, 73100 Lecce, Italy or carlogiacomo.leo@movementis.it (C.G.L.); egeria.scoditti@cnr.it (E.S.); 3 National Research Council, Institute for Applied Mathematics & Information Technologies ‘E. Magenes’, 20133 Milan, Italy; antonella.bodini@cnr.it; 4 Department of Pharmacy-Pharmaceutical Sciences, University of Bari Aldo Moro, 70125 Bari, Italy; francesco.bagordo@uniba.it; 5 MOVE-mentis s.r.l., 47522 Cesena, Italy; pierpaolo.mincarone@movementis.it; 6 Research Unit of Brindisi, National Research Council, Institute for Research on Population & Social Policies, 72100 Brindisi, Italy; 7 Oncology Unit, ‘Vito Fazzi’ Hospital, 73100 Lecce, Italy; dr.dematteis.elisabetta@gmail.com; 8 Department of Experimental Medicine, University of Salento, 73100 Lecce, Italy; tiziana.grassi@unisalento.it

**Keywords:** microRNA, biomarker, musculoskeletal pain, runners, bioinformatic analysis

## Abstract

**Background**: miRNAs are short, non-coding RNAs whose deregulation has been shown in painful processes, including musculoskeletal pain. This condition, which causes disability, impacts quality of life, and contributes to substantial healthcare costs, is also a critical issue in sports. In this case-control study, we evaluated the expression of four miRNAs involved in inflammation in runners with musculoskeletal pain and elucidated their functions and pathophysiological implications. **Methods**: A total of 17 runners with musculoskeletal pain and 17 age- and sex-matched runners without pain participated in this study. The levels of the miRNAs were evaluated by qRT-PCR. Bioinformatic tools were employed to identify the target genes and biological processes regulated by these miRNAs. **Results**: Compared to the controls, the runners with musculoskeletal pain exhibited significantly higher plasma levels of miR-133b (*p* = 0.02), miR-155-5p (*p* = 0.003) and let-7a-5p (*p* = 0.02). Multivariable regression analysis indicated that these three miRNAs exhibit a positive correlation (*p* < 0.05) with the presence of musculoskeletal pain, adjusted for age. Bioinformatic analysis suggested that the miRNAs hub genes are involved in regulatory processes, neuroinflammatory pathways, and human diseases that are associated with pain pathology. **Conclusions**: These results enhance our understanding of the potential role of miR-133b, miR-155-5p and let-7a-5p in pain-associated biological processes. The miRNA-mediated negative regulation of genes identified could explain the inflammatory and tissue repair processes in this population. Further studies are needed to confirm and validate the role of these miRNAs in painful conditions, especially considering the significant public health implications of managing inflammatory pain in sports.

## 1. Introduction

Musculoskeletal pain, defined as persistent or recurrent pain affecting muscles, tendons, ligaments and bones [1], is a significant health issue, afflicting approximately 1.71 billion people worldwide [2]. This condition not only contributes to substantial healthcare costs but also significantly impacts quality of life and productivity [3,4].

In the context of sports, musculoskeletal pain presents a critical challenge [5], as it can limit athletes’ training capacity and performance, potentially leading to a loss of competitiveness [6]. Athletes, particularly runners, frequently experience musculoskeletal pain, with the common sites including the lower back and the lower extremities. Such pain is often linked to repetitive mechanical stress, overuse, and inflammation, all of which may contribute to the onset of tissue damage [5,7].

Musculoskeletal pain involves multiple signaling pathways and molecular mediators [8,9]. Although its pathophysiology remains incompletely understood, the painful sensation is triggered by a noxious stimulus that transmits from the periphery, via a depolarization wave, through the spinal cord and into various areas of the central nervous system. At the site of injury or inflammation, inflammatory mediators—such as prostaglandins and cytokines (e.g., interleukin-6, tumor necrosis factor-alpha, and chemokines)—are released, initiating signaling pathways that activate or sensitize nociceptors, leading to pain generation [10,11]. These inflammatory mediators contribute to the sensitization of nociceptors by interacting with ion channels like TRPV1 and Nav1.7, which play critical roles in the transmission of pain signals [9].

Inflammatory mediators are influenced by a variety of bioactive molecules, which interact in complex ways. Many of these molecules contribute to both peripheral and central sensitization through synergistic or inhibitory interactions, creating a network that plays a role in the development of pain [12].

Recent research has highlighted the regulatory role of microRNAs (miRNAs) in pain signaling and inflammation [12,13,14]. MiRNAs are small, non-coding RNA molecules containing 20–22 nucleotides that modulate gene expression post-transcriptionally [15]. Their expression can be influenced by various lifestyle factors [16] and stress conditions [17]; moreover, these molecules play pivotal roles in physiological [18] and pathological processes [19,20].

MiRNAs are not only found intracellularly but also in extracellular fluids like blood, serum, plasma, and saliva (circulating miRNAs). Notably, circulating miRNAs exhibit remarkable stability and resistance to degradation [21], making them potential biomarkers in medical conditions, including pain.

Several miRNAs influence pain perception by modulating the expression of target genes involved in the pain signaling pathway, including nociceptors and spinal interneurons [22]. For instance, miR-146a-5p plays a key role in negatively regulating pro-inflammatory cytokine signaling via the NF-κB pathway, thus influencing the inflammatory response. It is upregulated in response to inflammatory stimuli and helps modulate this response by inhibiting cytokine production and affecting pain perception through targeting the mRNA of pro-inflammatory genes like *IRAK1* and *TRAF6* [23,24,25]. Another example is miR-155-5p, which has been shown to enhance the expression of inflammatory cytokines and amplify pain in various inflammatory conditions [26,27]. MiR-133b is essential for muscle regeneration, particularly in differentiating satellite cells for tissue repair; dysregulation of this miRNA may impair healing and contribute to chronic pain, making it a key factor in sports-related injuries [28,29]. Again, let-7a-5p has been shown to regulate inflammation and nociceptor sensitivity [30].

In athletes, where inflammation and mechanical stress are common, miRNAs may serve as important regulators of pain processing [31,32]. Understanding the interactions between miRNAs and their targets could provide insight into the mechanisms underlying pain and offer new therapeutic strategies for managing it [33,34].

Despite the advances in understanding the role of miRNAs in pain, several gaps remain, particularly regarding their exact roles in the context of sports-related musculoskeletal pain. We hypothesize that the miRNAs previously mentioned (hsa-miR-133b, hsa-miR-146a-5p, hsa-miR-155-5p, and hsa-let-7a-5p) as having been identified as being involved in pain processes [13,14] are deregulated in runners with musculoskeletal pain and may contribute to the modulation of the inflammatory and nociceptive pathways underlying pain perception.

The aim of this study was to evaluate the expression of these four miRNAs in paired plasma and saliva samples from long-distance runners with musculoskeletal pain compared to runners without pain. Multiple linear regression analysis was used to identify key predictors of plasma miRNA expression, assessing the influence of pain, age, physical activity and self-reported stress. Additionally, bioinformatic analysis was conducted to characterize the miRNAs’ function and their potential pathophysiological implications, providing insight into how these miRNAs may contribute to the regulation of pain in athletic contexts.

## 2. Materials and Methods

### 2.1. Study Design and Participants

This study was part of the MiMuS project (“microRNAs as biomarkers of musculoskeletal pain in long distance runners”), whose complete description was previously published [35]. In brief, the MiMuS project is a research initiative that encompasses both a case-control study and a quasi-experimental study.

It includes thirty-four long-distance runners affiliated with the Lecce sections of the Italian Federation of Athletics (FIDAL), aged 35 years or older, possessing a medical certificate for competitive sports activities. Participants with any of the following criteria were excluded: presence of chronic diseases; ongoing pregnancy or within 6 months post-childbirth; within 6 months post-weaning.

The study protocol was approved by the Ethics Committee of the Lecce Local Health Authority (ASL/LE) (deliberation no. 0000108, 2 February 2023).

The recruited subjects were administered both validated questionnaires (the short form of the International Physical Activity Questionnaire, IPAQ, the 11-point Numerical Rating Scale (NRS) for pain intensity, and the perceived stress questionnaire) [36,37,38] and questions specifically developed by the researchers to collect sociodemographic, lifestyle and anthropometric information. Participants with ongoing musculoskeletal pain who wished to take part in the study were first assigned to the cases group, while the control group (individuals without pain for at least one month) was selected using covariate balancing for age and sex. Both the questionnaire administration and plasma/saliva collection were performed at rest for both the cases and controls.

Following the sample collection, the cases group underwent a kinesiological intervention that avoided direct treatment of inflamed or painful areas and instead focused on correcting poor posture caused by a limited range of joint motion or by weak or tight muscles [39]. The analysis of the miRNA expression following the kinesiological intervention will be discussed in detail in a separate article.

### 2.2. Quantification of miRNAs

The miRNAs under study were chosen based on their known involvement in the pathogenesis of pain and inflammation [13,14], and they are hsa-miR-146a-5p (Assay ID: 000468), hsa-miR-155-5p (Assay ID: 467534_mat), hsa-miR-133b (Assay ID: 002085), and hsa-let-7a-5p (Assay ID: 000377). Fasting venous blood samples were obtained in the morning (from 8 to 10) from thirty-four runners to measure the miRNA expression levels using special vacutainer-type test tubes with ethylenediaminetetraacetic acid (EDTA) as an anticoagulant. The blood was processed to isolate the plasma by centrifugation at 1900× *g* for 10 min at 4 °C. The plasma was then aliquoted and stored at −80 °C until the RNA extraction.

Before the saliva collection, the same number of participants as for the blood samples were instructed to abstain from eating or smoking for a minimum of 2 h. Saliva was collected in Falcon tubes and then centrifuged at 3000× *g* for 15 min at 4 °C. The supernatant was carefully transferred into Eppendorf tubes and stored at −80 °C until further analysis.

The total RNA was extracted from the paired plasma and saliva samples of each participant using the Quick-cfRNA^TM^ Serum and Plasma Kit (Zymo Research, Orange, CA, USA), adhering to the manufacturer’s protocol. The RNA concentration and purity were measured with a NanoDrop spectrophotometer at wavelengths of 230, 260 and 280 nm.

Following this, cDNA was synthesized from the RNA using the TaqMan MicroRNA Reverse Transcription Kit (Thermo Fisher Scientific, Waltham, MA, USA). The RT mixture was incubated at 16 °C for 30 min, 42 °C for 30 min, and then at 85 °C for 5 min. The real-time quantitative PCR reactions was carried out using TaqMan Universal PCR Master Mix II along with specific TaqMan miRNA Assays (Applied Biosystems, Foster City, CA, USA). The PCR protocol included an initial denaturation at 95 °C for 10 min, followed by 45 cycles of denaturation at 95 °C for 15 sec and annealing/extension at 60 °C for 60 s. The expression levels of hsa-miR-16-5p (Assay ID: 000391) were utilized as endogenous reference controls to normalize the expression of the target miRNAs in each sample from both groups. This gene was chosen based on its demonstrated stable expression across all the samples analyzed. All the PCR reactions were conducted in duplicate for each sample.

The 2−ΔΔCt method was used to determine the fold change in the miRNA transcript levels [40].

### 2.3. Target Gene Prediction and Function and Pathway Enrichment Analysis

The target gene prediction for the dysregulated miRNAs was conducted using three databases, namely TargetScanVert [41], miRDB [42], which is accessible via miRbase [43], and miRWalk [44]. These databases were chosen due to their established reliability and comprehensive coverage of miRNA target predictions. By integrating the results from multiple databases, we aimed to enhance the robustness of our predictions, as each database utilizes distinct algorithms and datasets for target identification, reducing potential biases and improving the reliability of the targets identified.

To increase the robustness of the predictions, only target genes identified by all three databases were included in subsequent analyses. InteractiVenn [45] was employed as a tool to retrieve the overlapping target genes, allowing for a clear visualization of the common targets shared by the selected miRNAs.

To identify the hub genes, the predicted targets were analyzed using the STRING database (http://www.string-db.org/) to construct a protein–protein interaction (PPI) network [46]. PPIs with a confidence score ≥0.700 were imported into Cytoscape software (version 3.10.2) for further analysis [47]. The hub genes were identified using the CytoHubba plugin, applying multiple algorithms (Degree, Maximum Clique Centrality—MCC, Closeness, Maximum Neighborhood Component—MNC, Stress, Betweenness, Bottleneck, and Edge Penetration Component—EPC). The top hub genes were selected based on their overlap across these algorithms, and their connections with the miRNAs were analyzed using Cytoscape to highlight the most significant regulatory interactions.

For the Gene Ontology (GO) [48] and Kyoto Encyclopedia of Genes and Genomes (KEGG) pathway enrichment analysis [49], the top hub genes were analyzed using the Database for Annotation, Visualization and Integrated Discovery (DAVID) bioinformatics tool [50]. The GO analysis included the three main categories: Biological Process (BP), Molecular Function (MF), and Cellular Component (CC). The most relevant BP, MF, CC, and signaling pathways associated with the hub genes of the deregulated miRNAs were reported. A *p*-value < 0.05 and False Discovery Rate correction were applied to determine statistical significance.

### 2.4. Statistical Analysis

Given that the distribution of miRNA levels is typically non-Gaussian, descriptive analysis of these variables was presented as the median and 25–75% percentiles. The normality of the distribution of the other variables was assessed using the Shapiro–Wilk test. Normally distributed data were presented as the mean ± standard deviation. Categorical variables were described as the counts and percentages.

The differences in the miRNA expression between the subjects with pain and the controls were analyzed by the Mann–Whitney U test. As for the other variables, the *t*-test or the U test were used, based on the normality test. Correlation analysis was performed using the Spearman correlation coefficient. All the tests were two-sided. The significance level was set at 0.05.

For the miRNAs exhibiting a significant association with pain, multivariable linear regression analysis was carried out to determine whether the bivariate association persists when adjusting for covariates known to influence miRNA expression, such as age, BMI, physical activity, and self-reported stress. The model selection was carried out using the Akaike Information Criterion (AIC). The Box–Cox transformation was applied for non-Gaussian variables. If normality was not achieved, iteratively reweighted least squares robust regression analysis was also conducted [34]. This method weights each data point to reduce the influence of those with high residuals. The statistical analyses were conducted using the open-source software R 4.2.0 [51].

## 3. Results

### 3.1. Participants Characteristics

There were 17 participants (14 males and 3 females) in both the cases and controls groups. As shown in Table 1, there was a significant difference only for the stress level and total physical activity, which was higher in the cases group compared to the controls group. Table 1 summarizes these results.

In terms of lifestyle factors, including smoking and alcohol consumption, only one subject in the cases group was a current smoker, and two subjects in the cases group and one in the controls group reported abstaining from alcohol consumption. Therefore, these variables were not considered in the subsequent analyses.

Regarding the NRS for pain intensity, eight subjects reported an intensity level of four, while the other subjects reported higher levels. Additionally, most participants indicated that their pain was predominantly located in the knee and lower back (Table 2).

### 3.2. miRNA Quantification

**Figure 1:**
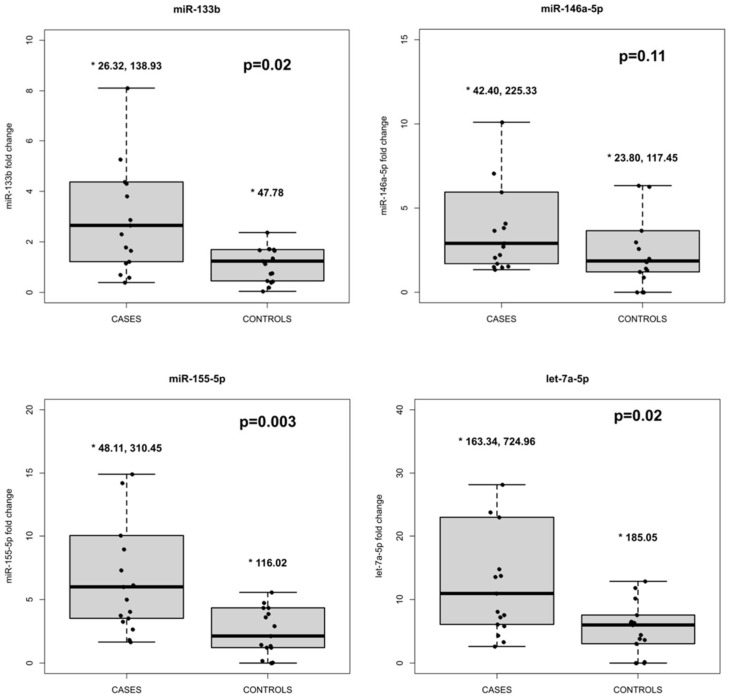
Boxplots comparing the expression levels of the plasma miRNAs between the cases group and the controls group. The *p*-value refers to the two-tailed Mann–Whitney U test. Outliers are indicated above each boxplot, not represented in the graph for reasons of clarity.

The relative expression levels of the four selected miRNAs in the plasma are shown in Figure 1. We found a significant difference between the cases and controls in terms of hsa-miR-133b (*p* = 0.02), hsa-miR-155-5p (*p* = 0.003), and hsa-let-7a-5p (*p* = 0.02), indicating upregulation for the cases. Although hsa-miR-146a-5p exhibited a higher fold change in the cases compared to the controls, the difference was not statistically significant (*p* = 0.11). In Table 3 are reported the estimated differences in location and their 95% CI.

We also conducted a similar analysis of the saliva samples to explore the potential role of using this non-invasive method for studying musculoskeletal pain. In this analysis, we found a significant difference for hsa-miR-155-5p (fold change cases: median 12.58, IQR: 6.38–26.06 vs. fold change controls: median 6.93, IQR: 0.05–12.76, *p* = 0.04), with overexpression observed in the cases group. Additionally, while overexpression was also observed in the cases group for the other miRNAs, these differences were not statistically significant: hsa-miR-133b (fold change cases: median 6.38 IQR: 1.99–17.44 vs. fold change controls: median 3.42, IQR: 0.10–6.38, *p* = 0.10), hsa-miR-146a-5p (fold change cases: median 1.39, IQR: 0.76–1.88 vs. fold change controls: median 1.1, IQR: 0.67–1.9, *p* = 0.48), and hsa-let-7a-5p (fold change cases: median 22.53, IQR: 14.36–57.03 vs. fold change controls: median 20.44, IQR: 0.02–42.92, *p* = 0.32) (Figure S1 in the Supplementary Materials).

### 3.3. Correlation Analysis

**Figure 2:**
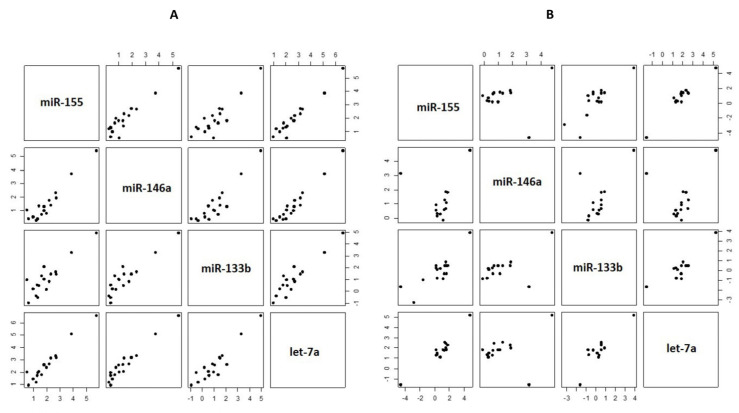
Pairwise scatterplot of the plasma miRNA expression in (**A**) the cases group; and (**B**) the control group. The data points were log-transformed to enhance the visualization.

The plasma miRNAs showed significant correlations with each other in the cases group (*p* < 0.0009) (Table 4 and Figure 2A). In the controls group, the significance of these correlations weakened for hsa-miR-133b when compared to hsa-mir-146a-5p and hsa-mir-155-5p (*p* < 0.11), while the other comparisons remained statistically significant (Table 5 and Figure 2B).

Additionally, we investigated the relationship between the plasma miRNAs and age, BMI, level of physical activity and perceived stress across all the subjects. Age was significantly correlated with hsa-miR-133b (ρ = −0.40, *p* = 0.03), hsa-miR-146a-5p (ρ = −0.37, *p* = 0.03), and hsa-let-7a-5p (ρ = −0.48, *p* = 0.009), while weak correlation was observed with hsa-miR-155-5p (ρ = −0.34, *p* = 0.07). No significant correlation was found with the BMI (−0.03 ≤ ρ ≤ 0.093, *p* > 0.53). Physical activity showed a weak correlation with hsa-miR-133b (ρ = 0.30, *p* = 0.08). The (binary) level of perceived stress was associated with all the plasma miRNAs, with significant associations observed for hsa-miR-133b and hsa-let-7a-5p (*p* < 0.04), while the other two miRNAs displayed weaker associations (*p* < 0.08). In all the cases, higher plasma miRNA levels were found in the subjects reporting a medium level of stress.

### 3.4. Regression Analysis

For hsa-miR-133b, hsa-miR-155-5p, and hsa-let-7a-5p, which exhibited a significant association with musculoskeletal pain, multivariable linear models of the plasma miRNA levels were conducted, using age, physical activity, and self-reported stress (binary) as predictors, based on the results of the correlation analysis. The selection of the model based on the AIC led to considering only the presence of pain and age as predictors for each of the miRNAs. However, the Box–Cox transformations did not allow the residuals to be normalized in any of the three cases. For this reason, Table 6 reports the coefficients estimated using the robust linear regression method. The analysis revealed that hsa-miR-133b, hsa-miR-155-5p and hsa-let-7a-5p exhibit a positive correlation (*p* < 0.05) with the presence of musculoskeletal pain, adjusted for age.

### 3.5. Identification of miRNA Target Genes and Hub Genes

The results of the case-control study were used to conduct a bioinformatics analysis aimed at identifying the gene targets of hsa-miR-133b, hsa-miR-155-5p, and hsa-let-7a-5p.

**Figure 3:**
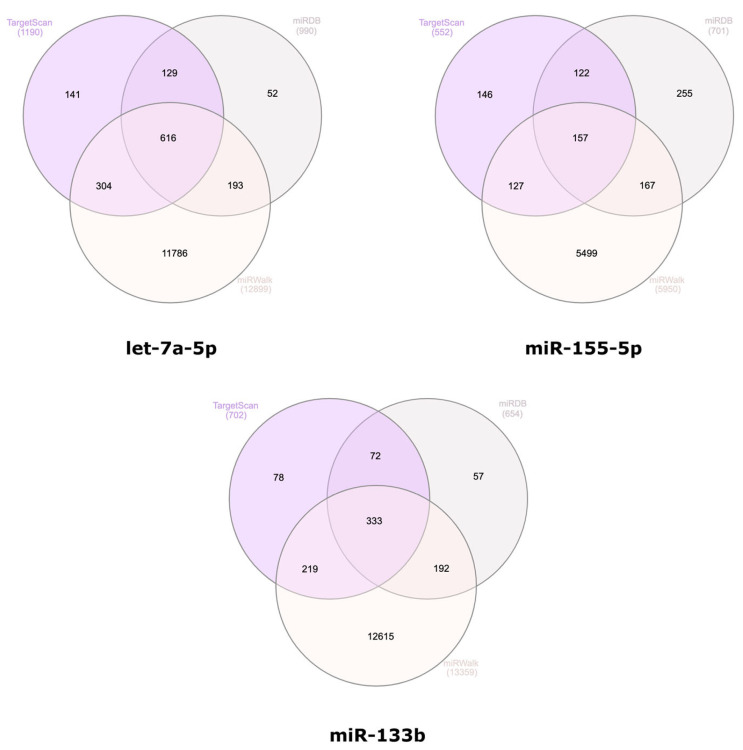
Venn diagram displaying the predicted target genes of the selected miRNAs, as sourced from three distinct databases.

Using InteractiVenn, the following overlapping genes were obtained: 616 targets for hsa-let-7a-5p, 157 targets for hsa-miR-155-5p, and 333 targets for hsa-miR-133b (Figure 3). Among the common target genes of hsa-miR-133b, hsa-miR-155-5p and hsa-let-7a-5p, adaptor-associated protein kinase 1 (AAK1) was identified.

**Figure 4:**
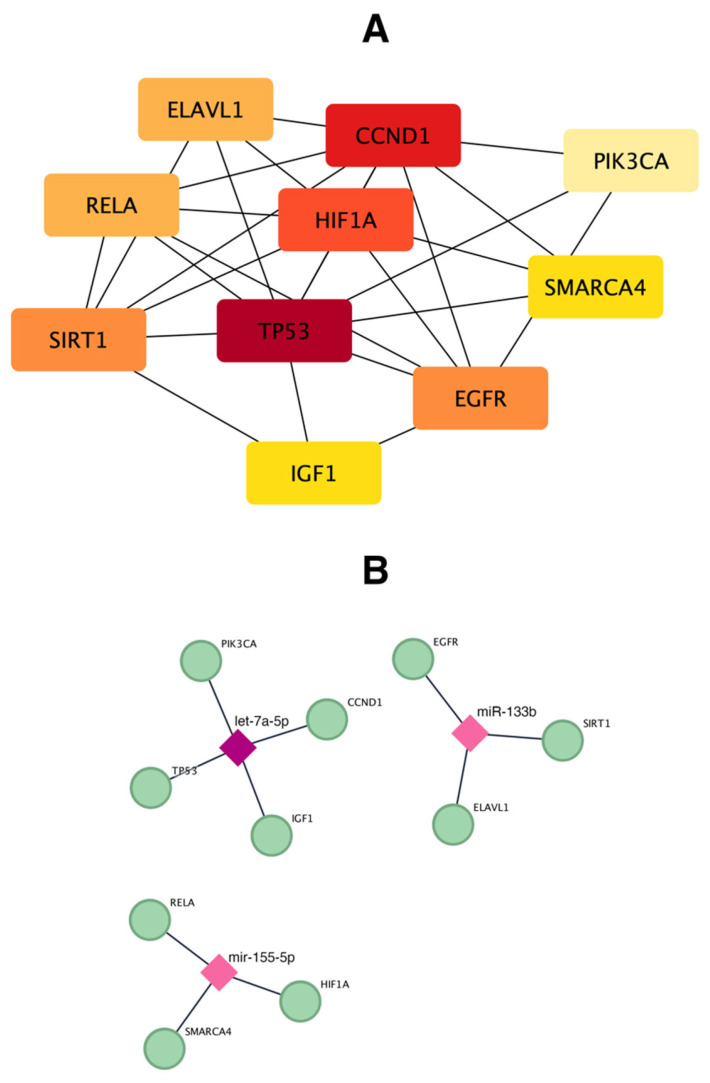
Network construction. (**A**) Identification of 10 hub genes determined through 8 algorithms in CytoHubba. A darker color (red) indicates higher connectivity among the hub genes. (**B**) Interaction network illustrating the relationship between the miRNAs and the 10 identified hub genes. Diamonds represent miRNAs, while ellipses represent the corresponding target genes. A darker color (dark pink) indicates a higher connectivity within the network.

To further analyze these targets, a PPI network was created using the STRING database. A total of 1046 nodes and 1188 edges were identified, reflecting the complex interactions among the target genes. To identify and analyze the key hub genes within the PPI network, the CytoHubba plugin of Cytoscape was utilized. By applying eight algorithms, the top 10 hub genes were selected based on their overlap across these algorithms. These include *TP53, SIRT1, RELA; PIK3CA, IGF1, HIF1A, ELAVL1, EGFR, CCND1,* and *SMARCA4*, highlighting the most significant regulatory interactions within the network (Table 7 and Figure 4A).

A network of the three miRNAs and the top 10 hub genes was then built using Cytoscape (Figure 4B). This network illustrates how these miRNAs potentially regulate the hub genes, which may play critical roles in the development and persistence of musculoskeletal pain in runners.

### 3.6. Function and Pathway Enrichment Analysis of Hub Genes

**Figure 5:**
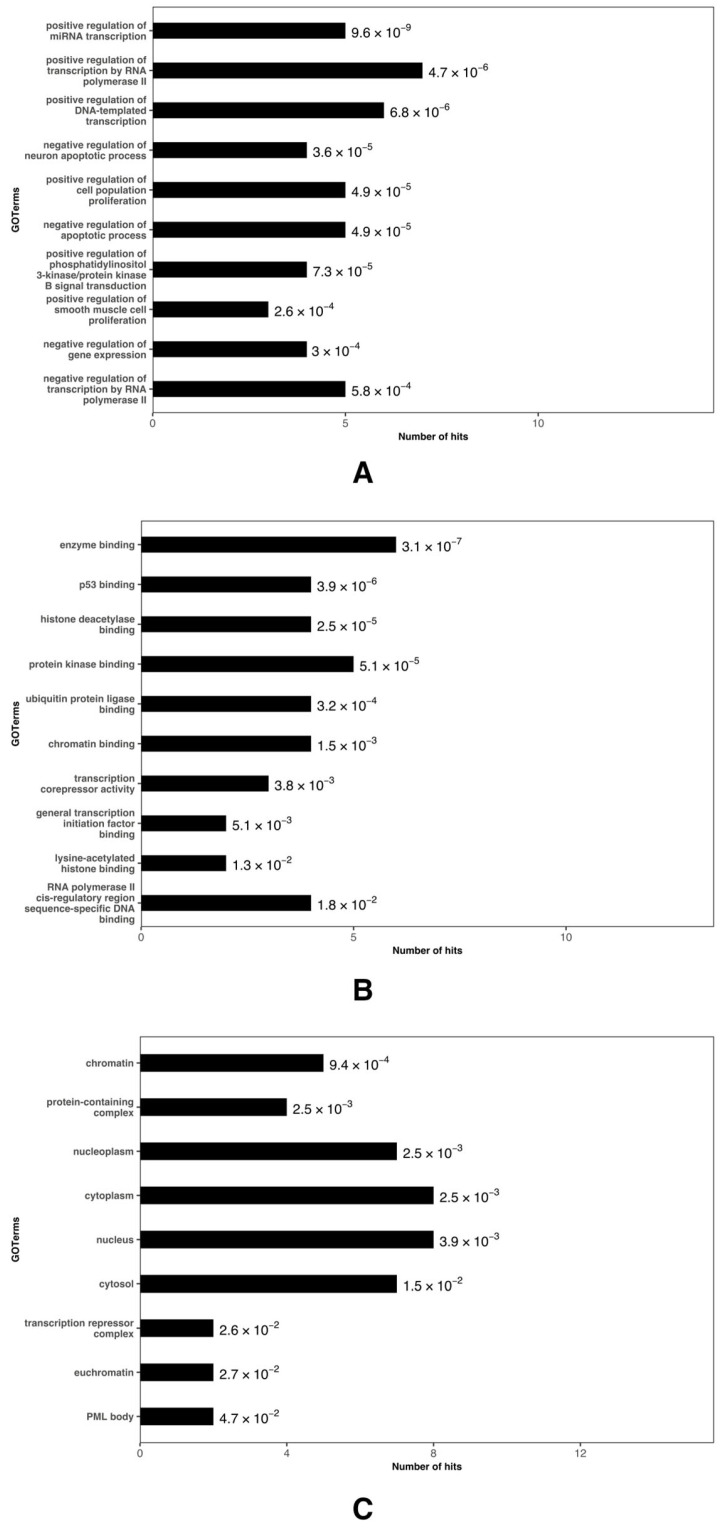
Gene Ontology (GO) analysis of the miRNA hub genes, depicting: (**A**) Biological Processes, (**B**) Molecular Functions, and (**C**) Cellular Components. The GO terms are arranged according to their *p*-values, in descending order.

The GO analysis revealed the involvement in key BPs such as the regulation of transcription, apoptotic processes, and cell proliferation (Figure 5A and Table S1). The MFs identified include binding to enzymes, p53, histone deacetylase, protein kinase, and chromatin (Figure 5B and Table S1). These genes are also located in CCs such as chromatin, the cytoplasm, nucleus, nucleoplasm, and transcription repressor complexes (Figure 5C and Table S1).

**Figure 6:**
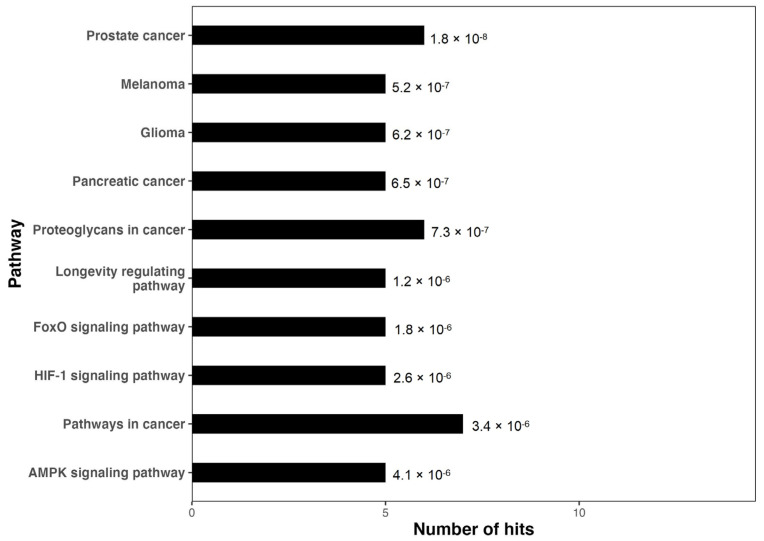
KEGG pathway enrichment analysis of the miRNA hub genes. The pathways are arranged in descending order based on the *p*-values, illustrating the significance of each pathway in relation to the identified hub genes.

KEGG pathway enrichment analyses of the top 10 hub genes were performed using DAVID. The 10 enrichment pathways identified through the KEGG pathway analysis were involved in signal transduction (e.g., FoxO signaling pathway, HIF-1 signaling pathway, AMPK signaling pathway), aging (e.g., longevity-regulating pathway), and human diseases (e.g., prostate cancer, melanoma, glioma, pancreatic cancer, etc.) (Figure 6 and Table S2).

## 4. Discussion

The present study examined the expression of four miRNAs with potential implications in terms of inflammatory pain [13,14] in runners experiencing musculoskeletal pain.

Our findings showed a significant upregulation of hsa-miR-133b, hsa-miR-155-5p and hsa-let-7a-5p in the runners with musculoskeletal pain compared to the controls, suggesting a possible role for these miRNAs in the pathogenesis of musculoskeletal pain. Their increased expression could reflect an alteration in the mechanisms regulating inflammation and pain in this population. Moreover, we found significant correlations among the miRNAs in the cases group, suggesting coordinated regulation under musculoskeletal pain conditions. This contrasts with the controls, where the correlation of hsa-miR-133b with hsa-miR-146a-5p and hsa-miR-155-5p was weaker. These differences may indicate that miRNAs interact more specifically and closely under pain conditions, potentially amplifying the pathological response associated with pain. Our results are consistent with previous studies that highlighted the involvement of these miRNAs in biological processes related to inflammation, pain and the response to muscle stress. For example, miR-155-5p is essential in the processes of inflammation and nociception acting through pathways that regulate the suppressor of cytokine signaling 1 (*SOCS1*) as well as the TNF-α receptor-transient receptor potential ankyrin 1 (TNFR1-TRPA1) pathways [27,52]. Its abnormal overexpression may act as a potential biomarker or therapeutic target for inflammatory conditions [53]. Our findings align with prior studies that demonstrated an association between miR-155-5p and pain conditions, including chronic low back pain, rheumatoid arthritis, and fibromyalgia [54,55].

Regarding let-7a-5p, it plays a role in cellular processes, inflammation, and immunity [56]. Let-7a-5p exhibits both anti-inflammatory and pro-inflammatory effects. For example, elevated let-7a-5p expression in microglia was associated with protection against ischemia and neuroinflammation by suppressing pro-inflammatory factors such as IL-6 and iNOS while enhancing the expression of anti-inflammatory agents like IL-10 and IL-4 [30]. Conversely, it may inhibit the immunomodulatory functions of bone marrow-derived mesenchymal stem cells, which can lead to a decrease in T-cell apoptosis and an enhancement of inflammatory responses [57].

MiR-133b is predominantly found in skeletal muscle and as such is categorized as a myomiR. Overexpression of this miRNA has also been observed in patients with muscular dystrophy compared to healthy individuals [58]. Additionally, increased levels of myomiRs have been documented in healthy individuals following marathon running [29,59]. Furthermore, studies have indicated that the discriminatory capability of myomiRs to distinguish between damaged and non-damaged muscle is superior to that of creatine kinase, suggesting that myomiRs may provide a more accurate means of diagnosing muscle damage [60].

Although miR-146a-5p did not reach statistical significance, it showed an increasing trend in the cases group, indicating its potential involvement in the pathogenic process of musculoskeletal pain. miR-146a-5p is known for its anti-inflammatory properties [61]. In response to pain and inflammation, the body often activates anti-inflammatory mechanisms to mitigate tissue damage and facilitate recovery. Thus, the overexpression of miR-146a-5p could represent an adaptive response to attenuate inflammation and alleviate musculoskeletal pain. Nevertheless, this trend warrants consideration in future studies with larger samples.

By comparing the expression profiles of the miRNAs in plasma and saliva, we identified that the analyzed miRNAs are expressed in both biofluids, albeit with unique expression patterns in each. Further studies with larger sample sizes are needed to better elucidate the significance of the miRNA expression in saliva samples and to provide stronger conclusions regarding their potential as non-invasive source for studying pain-related miRNAs.

We also explored the relationship between the plasma miRNAs and factors such as age, BMI, physical activity, and perceived stress. Significant correlations were found between age and miR-133b and let-7a-5p, consistent with previous studies highlighting age-related changes in miRNA expression [62,63,64]. In contrast, the BMI showed no significant association with any of the miRNAs, while physical activity only weakly correlated with miR-133b, suggesting that these variables may not heavily influence miRNA expression in this population. Interestingly, perceived stress, which was higher among the cases than the controls, was significantly associated with miR-133b and let-7a-5p, with greater expression levels observed in subjects reporting moderate stress. This confirms a potential link between psychological stress and miRNA modulation, as reported previously [17,65], possibly contributing to or amplifying inflammatory processes in individuals with musculoskeletal pain.

Based on the results from the correlation analysis, we performed a multivariable regression analysis, which revealed that miR-133b, miR-155-5p and let-7a-5p exhibit a notable positive correlation with musculoskeletal pain, suggesting that higher levels of these miRNAs may be associated with an increased risk of developing musculoskeletal pain in runners. Additionally, only hsa-let-7a-5p showed a significant negative correlation with age, in agreement with other studies indicating age-related changes in miRNA expression [64] and, in particular, an inverse correlation between let-7a-5p and advancing age [62,63]. However, it is important to note that these associations may be influenced by various confounding factors not accounted for in our analysis and should be further investigated in a larger population.

To better highlight the role of miRNAs significantly overexpressed in runners with pain, we conducted a bioinformatics analysis to identify the target genes and pathways involved in pain regulation. Our results showed that hsa-miR-133b, hsa-miR-155-5p and hsa-let-7a-5p share *AAK1* as a common target. *AAK1*, a Ser/Thr protein kinase [66], is involved in regulating several cellular signaling pathways, including those related to pain transmission and neuronal sensitization [67]. This suggests that these miRNAs could influence pain perception and musculoskeletal pain sensitivity by modulating AAK1 expression. We also identified 10 hub genes, including *TP53, SIRT1*, *RELA; PIK3CA*, *IGF1*, *HIF1A*, *ELAVL1*, *EGFR*, *CCND1*, and *SMARCA4*, which are involved in critical processes such as inflammation, pain modulation, and cellular stress responses. For instance, *TP53* regulates apoptosis and cell cycle processes crucial for tissue repair [68], while *SIRT1* and *HIF1A* are implicated in stress responses and hypoxia [69], conditions often linked to chronic pain. *ELAVL1* encodes an RNA-binding protein that stabilizes the mRNA of different inflammatory mediators and plays a crucial role in amplifying inflammatory signals [70]. *RELA***,** part of the NF-κB pathway, and *IGF1* are key regulators of inflammatory signaling and tissue repair, potentially affecting pain sensitivity and musculoskeletal function [71,72]. Notably, miR-155-5p has been reported to regulate RELA [73], let-7a-5p targets IGF1 and CCND1 [74], both important in tissue regeneration, and miR-133b has been implicated in the regulation of SIRT1 [75]. These findings suggest that these miRNAs might exert broad effects by regulating multiple hub genes associated with pain transmission and inflammation.

The GO analysis highlighted the involvement of these hub genes in essential biological processes like transcription regulation, apoptosis, and cell proliferation. These functions are particularly relevant in the context of chronic pain and inflammation, as the regulation of apoptosis and cell proliferation may impact tissue maintenance and the response to muscle injury [76,77]. Notably, the negative regulation of neuronal apoptosis and the positive regulation of transcription may indicate a neuroprotective role for these genes, reducing pain sensitivity in musculoskeletal tissues. Regarding the molecular functions, the association of these genes with p53, histone deacetylases, and other regulatory proteins indicates their influence on gene transcription and epigenetic modification, processes crucial for cellular adaptation to physical stress and inflammation during exercise [78,79]. Their localization in transcription repressor complexes and nuclear components, such as chromatin, reinforces the idea that these genes finely regulate gene expression and genome organization in response to mechanical and inflammatory stimuli [80].

The KEGG analysis revealed key signaling pathways such as FoxO, HIF-1, and AMPK, which are involved in oxidative stress, energy metabolism, and inflammation, all of which are critical processes in the context of musculoskeletal pain. The FoxO pathway, for instance, regulates cell survival and the response to tissue damage [81,82], while the HIF-1 pathway is crucial for adapting to hypoxia, a condition common in overworked muscles [83,84]. The AMPK pathway’s role in maintaining energy balance and reducing inflammation further underscores the importance of cellular metabolism in pain modulation [85]. Moreover, the enrichment of pathways related to human diseases, such as prostate cancer, melanoma, and glioma, suggests a potential overlap between cancer regulation and muscle damage responses. Although these pathways are typically studied in oncological contexts, they may share mechanisms related to cell survival and proliferation in response to tissue stress [86]. The role of proteoglycans, essential for the structure and function of the extracellular matrix, further links these findings to the inflammatory processes implicated in pain pathogenesis [87].

In summary, these findings suggest that miR-133b, miR-155-5p, and let-7a-5p may act as key regulators of musculoskeletal pain through their influence on hub genes and critical signaling pathways. This lays the foundation for further experimental validation and the exploration of potential therapeutic interventions targeting these miRNAs.

Our study has several limitations. First, the sample size is small. The literature lacks effect size estimates based on substantive data from which to derive a power-based sample size calculation. Some studies [54,88,89] recommend recruiting no fewer than 30 subjects in the cases group, perhaps because 30 is a commonly accepted rule of thumb for applying the *t*-test, even when the sample distribution is not Gaussian. In our study, this threshold was not met; however, non-parametric or robust methods were applied to account for the non-Gaussian distribution and small sample size. Future studies with larger cohorts and longitudinal designs are needed to further investigate the mechanistic links between miRNA expression and musculoskeletal pain, as well as the potential of miRNAs as biological markers of pain. The small simple size also limited our ability to assess whether different levels of miRNA expression are associated with varying pain intensities. Additionally, the near absence of female participants could be another limitation, considering that the intensity of pain changes depending on sex. Lastly, further in vitro or in vivo studies are necessary to validate the miRNA/mRNA interactions and assess their clinical relevance.

## 5. Conclusions

The overexpression of hsa-miR-133b, hsa-miR-155-5p and hsa-let-7a-5p in runners with musculoskeletal pain strengthens our understanding of their potential involvement in pain-associated biological processes. Moreover, the miRNA-mediated negative regulation of the genes identified through the bioinformatic analysis could explain the inflammatory and tissue repair processes in this population. These findings shed new light on the pathophysiology of musculoskeletal pain in runners and hold promise for the development of targeted therapeutic interventions. By understanding the role of miRNAs and their regulatory mechanisms, new strategies can be devised to prevent and treat pain, ultimately enhancing the health and performance of athletes. Finally, applying a screening of miRNAs known for being associated with musculoskeletal pain could help with the early identification of runners at higher risk of developing musculoskeletal pain and consequently allow for preventive measures and more personalized training programs.

The results of our research support the planning of future studies with larger sample sizes, which are needed to confirm and validate the role of these miRNAs in painful conditions, especially considering that the management of inflammatory pain in sports is also a critical issue from a public health perspective.

## Figures and Tables

**Table 1:** Participants characteristics.

Characteristics	Cases (n = 17)	Controls (n = 17)	*p* Value
Age years, mean ± SD	50.9 ± 7.45	52.7 ± 8.3	0.504 ^a^
BMI (kg/m^2^), mean ± SD	23.6 ± 2.3	24 ± 2.4	0.291 ^a^
Stress level, n (%)			0.014 ^b^*
Low	6 (35.3%)	14 (82.4%)	
Medium	11(64.7%)	3 (17.6%)	
Physical activity (METs/min/week): Total, median (25–75%)	10,102 (8445–12240)	6030 (3066–7080)	0.003 ^c^*

Categorical data are presented as number and percentage. Continuous data are presented as either mean ± standard deviation or median and 25–75% percentile, based on Gaussianity. ^a^ *t*-test, ^b^ Chi-Square, ^c^ Mann–Whitney U test * *p* < 0.05.

**Table 2:** Pain sites.

Pain Sites	Frequencies
Lower back, knee	12.5%
Pelvis, thigh	9.4%
Calf, Achilles tendon, tibialis anterior, ankles, hamstring, gluteus, shoulder	6.3%
Foot, hip, adductor, piriform	3.1%

**Table 3:** Estimated differences in the location of the plasma miRNAs between cases and controls with 95% confidence intervals.

	Estimated Difference in Location ^1^	Lower-Bound 95%CI	Upper-Bound 95%CI
hsa-miR-133b	1.329639	0.2197724	3.0595326
hsa-miR-146a-5p	1.089028	−0.3309437	2.8595996
hsa-miR-155-5p	3.418816	1.389315	6.860126
hsa-let-7a-5p	5.859823	1.071484	13.350781

^1^ The estimator for the difference in the location parameters estimated the median of the difference between a sample from the cases group and a sample from the controls group.

**Table 4:** Spearman correlation coefficients between the plasma miRNAs in the cases group.

Case Group	hsa-mir-133b	hsa-mir-146a-5p	hsa-mir-155-5p	hsa-let-7a-5p
hsa-mir-133b	1	0.8064 *	0.7426 *	0.8333 *
hsa-mir-146a-5p		1	0.8235 *	0.9363 *
hsa-mir-155-5p			1	0.9240 *
hsa-let-7a-5p				1

* *p* < 0.0009.

**Table 5:** Spearman correlation coefficients between the plasma miRNAs in the controls group.

Control Group	hsa-mir-133b	hsa-mir-146a-5p	hsa-mir-155-5p	hsa-let-7a-5p
hsa-mir-133b	1	0.4585 °	0.4368 °	0.5753 *
hsa-mir-146a-5p		1	0.6036 *	0.7020 ^#^
hsa-mir-155-5p			1	0.8998 ^#^
hsa-let-7a-5p				1

* *p* < 0.05; ^#^ *p* < 0.003; ° *p* < 0.11.

**Table 6:** Plasma samples: estimated coefficients, standard errors and *p*-values of the selected models, based on robust linear regression.

Variables	hsa-miR-155-5p			hsa-miR-133b			hsa-let-7a-5p		
	Estimated Coefficient	Std. Error	*p*	Estimated Coefficient	Std. Error	*p*	Estimated Coefficient	Std. Error	*p*
Intercept	9.9809	4.4932	0.03 *	4.4697	2.0072	0.03 *	27.5882	6.9574	*p* < 0.001 *
Presence of pain	3.4562	1.2835	0.01 *	1.4172	0.5734	0.02 *	4.2873	1.9874	0.04 *
Age	−0.1383	0.0835	0.11	−0.0605	0.0373	0.11	−0.4110	0.1293	0.003 *

* *p* < 0.05.

**Table 7:** Top 20 ranked hub genes using CytoHubba.

Degree	Bottleneck	MCC	Stress	Closeness	EPC	MNC	Betweenness	Overlap
*TP53*	*TP53*	*TP53*	*TP53*	*TP53*	*RELA*	*TP53*	*TP53*	*TP53*
*EGFR*	*EGFR*	*CCND1*	*EGFR*	*EGFR*	*IGF1*	*EGFR*	*EGFR*	
*CCND1*	*RELA*	*EGFR*	*ELAVL1*	*CCND1*	*EGFR*	*CCND1*	*HIF1A*	*EGFR*
*PIK3CA*	*IGF1*	*RELA*	*SIRT1*	*HIF1A*	*IGF1R*	*KRAS*	*GNAS*	
*KRAS*	*ANK2*	*MAPK8*	*GABARAPL1*	*FOS*	*TP53*	*PIK3CA*	*ANK2*	*CCND1*
*FOS*	*SIRT1*	*CDKN1A*	*RELA*	*RELA*	*FOS*	*NRAS*	*ELAVL1*	
*RELA*	*CCND1*	*FOS*	*HIF1A*	*SIRT1*	*CCND1*	*IGF1*	*RELA*	*PIK3CA*
*HIF1A*	*HIF1A*	*SIRT1*	*CCND1*	*IGF1R*	*SIRT1*	*SMAD2*	*SIRT1*	
*NRAS*	*ELAVL1*	*BCL2L1*	*SMAD2*	*KRAS*	*KRAS*	*ELAVL1*	*GABARAPL1*	*RELA*
*SMARCA4*	*GNAS*	*KRAS*	*ANK2*	*SMAD2*	*NRAS*	*SMARCA4*	*SMAD2*	
*IGF1*	*SMARCA4*	*HIF1A*	*SMARCA4*	*PIK3CA*	*HIF1A*	*FOS*	*SMARCA4*	*HIF1A*
*SMAD2*	*SYT1*	*NRAS*	*KRAS*	*NRAS*	*PIK3CA*	*SIRT1*	*RCOR1*	
*SIRT1*	*FGF1*	*PIK3CA*	*GNAS*	*IGF1*	*SMAD2*	*HIF1A*	*UBXN7*	*SMARCA4*
*ELAVL1*	*GABARAPL1*	*IGF1R*	*IGF1*	*MAPK8*	*SMARCA4*	*MAPK8*	*SYT1*	
*MAPK8*	*THBS1*	*IGF1*	*IGF1R*	*SMARCA4*	*RPS6KB1*	*CDKN1A*	*DICER1*	*IGF1*
*IGF1R*	*BNIP3L*	*ELAVL1*	*EPHA4*	*RPS6KB1*	*ELAVL1*	*RELA*	*IGF1*	
*ANK2*	*PIK3CA*	*SMARCA4*	*PIK3CA*	*SP1*	*SP1*	*SP1*	*FGF1*	*SIRT1*
*SP1*	*NRAS*	*INSR*	*STX17*	*BCL2L1*	*MAPK8*	*CHD4*	*CCND1*	
*CDKN1A*	*SMAD2*	*RPS6KB1*	*ARRB1*	*GNAS*	*BCL2L1*	*RPS6KB1*	*PIK3CA*	*ELAVL1*
*RCOR1*	*RCOR1*	*GNAS*	*FGFR1*	*ELAVL1*	*INSR*	*BCL2L1*	*STX17*	

## Data Availability

The data presented in this study are available on request from the corresponding author due to the protection of participant confidentiality.

## Supplementary Materials

The following supporting information can be downloaded at https://www.mdpi.com/article/10.3390/biomedicines12112494/s1, Figure S1: Boxplots comparing the saliva miRNAs in the cases group versus the controls group. The *p*-value refers to the two-tailed Mann–Whitney test. Table S1: Significantly enriched GO terms of the hub genes of the miRNAs (the GO terms are arranged according to their *p*-values, in descending order). Table S2: Significantly enriched KEGG pathway terms of the target genes of the miRNAs (the pathways are arranged according to their *p*-values, in descending order).
