# Supplementary material for: MiRNA Expression in Long-Distance Runners with Musculoskeletal Pain: Implications for Pain Pathophysiology
Source: Biomedicines. 2024 Oct 30;12(11):2494. doi: 10.3390/biomedicines12112494 (PMC11591860; doi:10.3390/biomedicines12112494)
Supplement: Supplementary file 1 [file biomedicines-12-02494-s001.zip › biomedicines-3227047-supplementary.pdf]

Figure S1: Boxplots comparing the saliva miRNAs in the cases group versus the controls group. The p-value refers to the two-tailed Mann–Whitney test.

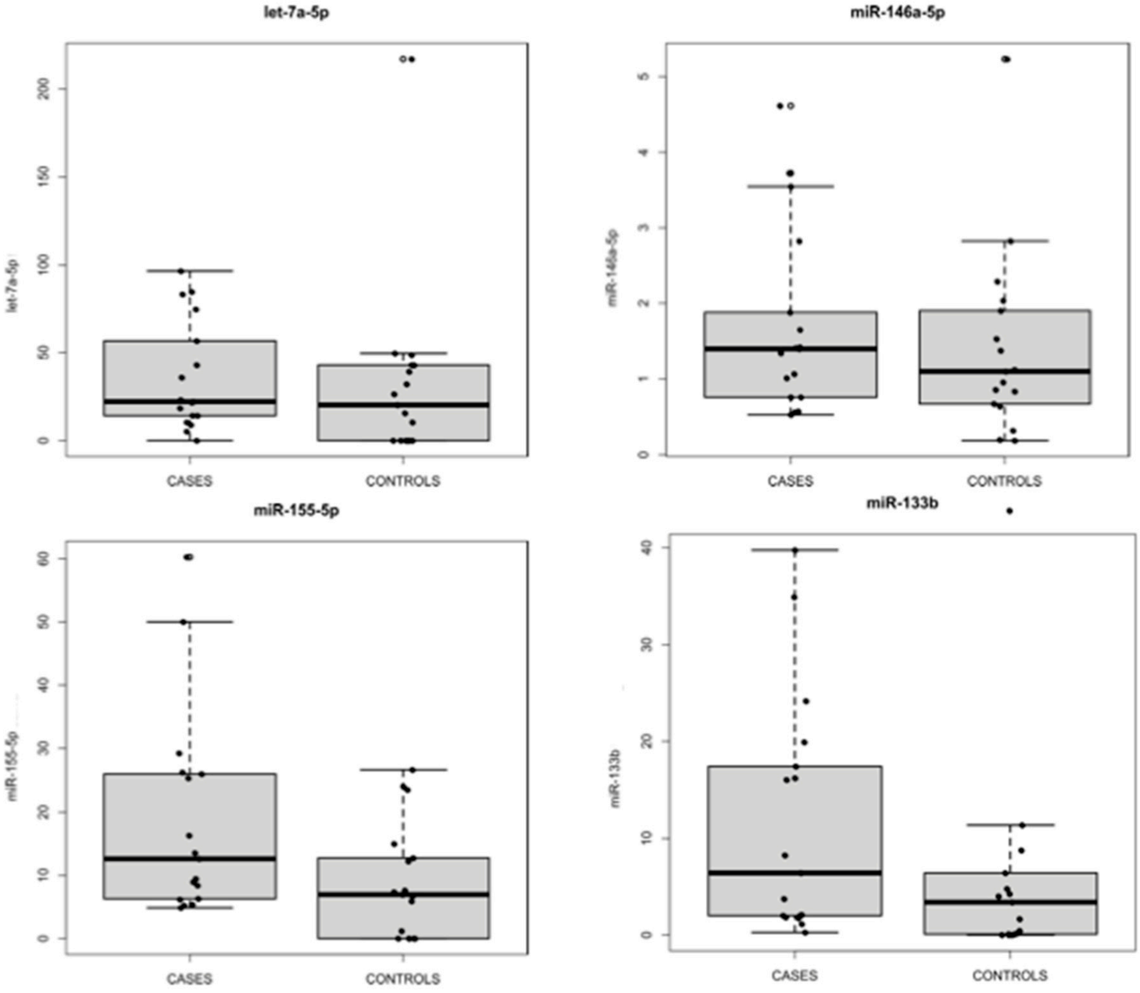

Table S1: Significantly enriched GO terms of the hub genes of the miRNAs (the GO terms are arranged according to their p-values, in descending order).

GO	GO ID	GENES	P-value	FDR
**Biological process**				
Positive regulation of miRNA transcription	1902895	*EGFR, HIF1A, RELA, SMARCA4, TP53*	9,60E-09	5,20E-06
Positive regulation of transcription by RNA polymerase II	0045944	*EGFR, HIF1A, IGF1, RELA, SIRT1, SMARCA4, TP53*	4,70E-06	1,20E-03
Positive regulation of DNA-templated transcription	0045893	*EGFR, HIF1A, IGF1, RELA, SMARCA4, TP53*	6,80E-06	1,20E-03
Negative regulation of neuron apoptotic process	0043524	*CCND1, HIF1A, PIK3CA, SIRT1*	3,60E-05	4,40E-03
Positive regulation of cell population proliferation	0008284	*EGFR, IGF1, SIRT1, SMARCA4, RELA*	4,90E-05	4,40E-03
Negative regulation of apoptotic process	0043066	*EGFR, IGF1, RELA, SIRT1, TP53*	4,90E-05	4,40E-03
Positive regulation of phosphatidylinositol 3-kinase/protein kinase B signal transduction	0051897	*EGFR, IGF1, PIK3CA, SIRT1*	7,30E-05	5,60E-03
Positive regulation of smooth muscle cell proliferation	0048661	*EGFR, IGF1, PIK3CA*	2,60E-04	1,80E-02
Negative regulation of gene expression	0010629	*HIF1A, IGF1, PIK3CA, SIRT1*	3,00E-04	1,80E-02
Negative regulation of transcription by RNA polymerase II	0000122	*CCND1, SIRT1, SMARCA4, RELA, TP53*	5,80E-04	3,10E-02
**Molecular function**				
Enzyme binding	0019899	*CCND1, EGFR, HIF1A, RELA, SIRT1, TP53*	3,10E-07	3,60E-05
p53 binding	0002039	*HIF1A, SIRT1, SMARCA4, TP53*	3,90E-06	2,30E-04
Histone deacetylase binding	0042826	*CCND1, HIF1A, RELA, TP53*	2,50E-05	9,90E-04
Protein kinase binding	0019901	*CCND1, EGFR, ELAVL1, HIF1A, RELA*	5,10E-05	1,50E-03
Ubiquitin protein ligase binding	0031625	*EGFR, HIF1A, RELA, TP53*	3,20E-04	7,60E-03
Chromatin binding	0003682	*EGFR, RELA, SMARCA4, TP53*	1,50E-03	2,90E-02
Transcription corepressor activity	0003714	*CCND1, SIRT1, SMARCA4*	3,80E-03	6,40E-02
General transcription initiation factor binding	0140296	*RELA, TP53*	5,10E-03	7,50E-02
Lysine-acetylated histone binding	0070577	*SIRT1, SMARCA4*	1,30E-02	1,60E-01
RNA polymerase II cis-regulatory region sequence-specific DNA binding	0000978	*HIF1A, RELA, SIRT1, TP53*	1,80E-02	1,90E-01
**Cellular component**				
Chromatin	0000785	*HIF1A, RELA, SIRT1, SMARCA4, TP53*	9,40E-04	5,30E-02
Cytoplasm	0005737	*CCND1, EGFR, ELAVL1, HIF1A, PIK3CA, RELA, SIRT1, TP53*	2,50E-03	5,30E-02
Nucleoplasm	0005654	*CCND1, ELAVL1, HIF1A, RELA, SIRT1, SMARCA4, TP53*	2,50E-03	5,30E-02
Protein-containing complex	0032991	*EGFR, IGF1, SMARCA4, TP53*	2,50E-03	5,30E-02
Nucleus	0005634	*CCND1, EGFR, ELAVL1, HIF1A, RELA, SIRT1, SMARCA4, TP53*	3,90E-03	6,60E-02
Cytosol	0005829	*CCND1, ELAVL1, HIF1A, PIK3CA, RELA, SIRT1, TP53*	1,50E-02	2,10E-01
Transcription repressor complex	0017053	*CCND1, TP53*	2,60E-02	2,90E-01

Euchromatin	0000791	*HIF1A, SIRT1*	2,70E-02	2,90E-01
PML body	0016605	*SIRT1, TP53*	4,70E-02	4,40E-01

Table S2: Significantly enriched KEGG pathway terms of the target genes of the miRNAs (the pathways are arranged according to their p-values, in descending order).

KEGG pathway	KEGG ID	GENES	P-value
Prostate cancer	hsa05215	*cyclin D1 (CCND1)*<br>*epidermal growth factor receptor (EGFR)*<br>*insulin-like growth factor 1 (IGF1)*<br>*phosphatidylinositol-4,5-bisphosphate 3-kinase catalytic subunit alpha (PIK3CA)*<br>*RELA proto-oncogene, NF-kB subunit (RELA)*<br>*tumor protein p53 (TP53)*	1,80E-08
Melanoma	hsa05218	*cyclin D1 (CCND1)*<br>*epidermal growth factor receptor (EGFR)*<br>*insulin-like growth factor 1 (IGF1)*<br>*phosphatidylinositol-4,5-bisphosphate 3-kinase catalytic subunit alpha (PIK3CA)*<br>*tumor protein p53 (TP53)*	5,20E-07
Glioma	hsa05214	*cyclin D1 (CCND1)*<br>*epidermal growth factor receptor (EGFR)*<br>*insulin-like growth factor 1 (IGF1)*<br>*phosphatidylinositol-4,5-bisphosphate 3-kinase catalytic subunit alpha (PIK3CA)*<br>*tumor protein p53 (TP53)*	6,20E-07
Pancreatic cancer	hsa05212	*cyclin D1 (CCND1)*<br>*epidermal growth factor receptor (EGFR)*<br>*phosphatidylinositol-4,5-bisphosphate 3-kinase catalytic subunit alpha (PIK3CA)*<br>*RELA proto-oncogene, NF-kB subunit (RELA)*<br>*tumor protein p53 (TP53)*	6,50E-07
Proteoglycans in cancer	hsa05205	*cyclin D1 (CCND1)*<br>*epidermal growth factor receptor (EGFR)*<br>*insulin-like growth factor 1 (IGF1)*<br>*hypoxia inducible factor 1 subunit alpha (HIF1A)*<br>*phosphatidylinositol-4,5-bisphosphate 3-kinase catalytic subunit alpha (PIK3CA)*<br>*tumor protein p53 (TP53)*	7,30E-07
Longevity regulating pathway	hsa04211	*insulin-like growth factor 1 (IGF1)*<br>*phosphatidylinositol-4,5-bisphosphate 3-kinase catalytic subunit alpha (PIK3CA)*<br>*RELA proto-oncogene, NF-kB subunit (RELA)*<br>*sirtuin 1 (SIRT1)*<br>*tumor protein p53 (TP53)*	1,20E-06
FoxO signaling pathway	hsa04068	*cyclin D1 (CCND1)*<br>*epidermal growth factor receptor (EGFR)*<br>*insulin-like growth factor 1 (IGF1)*<br>*phosphatidylinositol-4,5-bisphosphate 3-kinase catalytic subunit alpha (PIK3CA)*<br>*sirtuin 1 (SIRT1)*	1,80E-06
HIF1-1 signaling pathway	hsa04066	*epidermal growth factor receptor (EGFR)*<br>*hypoxia inducible factor 1 subunit alpha (HIF1A)*<br>*insulin-like growth factor 1 (IGF1)*<br>*phosphatidylinositol-4,5-bisphosphate 3-kinase catalytic subunit alpha (PIK3CA)*	2,60E-06

		RELA proto-oncogene, NF-kB subunit (RELA)	
Pathways in cancer	hsa05200	*cyclin D1 (CCND1)*<br>*epidermal growth factor receptor (EGFR)*<br>*hypoxia inducible factor 1 subunit alpha (HIF1A)*<br>*insulin-like growth factor 1 (IGF1)*<br>*phosphatidylinositol-4,5-bisphosphate 3-kinase catalytic subunit alpha (PIK3CA)*<br>*RELA proto-oncogene, NF-kB subunit (RELA)*<br>*tumor protein p53 (TP53)*	3,40E-06
AMPK signaling pathway	hsa04152	*cyclin D1 (CCND1)*<br>*ELAV-like RNA binding protein 1(ELAVL1)*<br>*insulin-like growth factor 1 (IGF1)*<br>*phosphatidylinositol-4,5-bisphosphate 3-kinase catalytic subunit alpha (PIK3CA)*<br>*sirtuin 1 (SIRT1)*	4,10E-06
